# Neuron-Specific Enolase and Hemoglobin as Risk Factors of Intraocular Metastasis in Patients with Renal Cell Carcinoma

**DOI:** 10.1155/2022/2883029

**Published:** 2022-04-23

**Authors:** Qiu-Yu Li, Ting Su, Wen-Qing Shi, Jian-Wen Fang, Meng-Yao Zhang, Qian-Hui Xu, Rong-Bin Liang, Qian-Min Ge, Biao Li, Yi Shao

**Affiliations:** ^1^Department of Ophthalmology, The First Affiliated Hospital of Nanchang University, Jiangxi Branch of National Clinical Research Center for Ocular Disease, Nanchang 330006, China; ^2^Eye Institute of Xiamen University, Fujian Provincial Key Laboratory of Ophthalmology and Visual Science, Xiamen 361102, China; ^3^Department of Ophthalmology, Massachusetts Eye and Ear, Harvard Medical School, Boston 02114, USA; ^4^Department of Breast Surgery, The First Affiliated Hospital, Zhejiang University School of Medicine, Hangzhou 310000, China

## Abstract

Renal cell carcinoma (RCC) appears to be a high risk of spread. This research investigated the correlation between a different range of clinical features and intraocular metastasis (IOM) in RCC patients and attempted to determine potential risk factors of RCC patients with IOM. In the study, there are a total of 351 patients with RCC that were recruited between May 1994 and May 2016. The differences between RCC patients with IOM and RCC patients with non-IOM (NIOM) were evaluated by the chi-squared test and Student *t* test. Binary logistic regression analysis was applied to determine risk factors. Finally, the value of diagnosis for RCC patients with IOM was assessed by receiver operating characteristic (ROC) curve analysis. Eighteen individuals were identified with IOM. There were no significant differences that were detected in alkaline phosphatase (AFP), carcinoembryonic antigen (CEA), alkaline phosphatase (ALP), cancer antigen 125 (CA-125), cancer antigen 153 (CA-153), cancer antigen 199 (CA-199), calcium, age, primary tumor site, and histopathological subtypes between the two groups. But there was a difference in terms of gender (*P* < 0.05). The IOM group exhibited significantly higher neuron-specific enolase (NSE) and lower hemoglobin (Hb) values compared to the NIOM group (*P* < 0.05, respectively). Binary logistic regression identified NSE and Hb as significant risk factors of IOM for RCC patient (*P* < 0.05 and *P* < 0.001, respectively). The ROC curve analysis indicated that the area under the curve (AUC) values of NSE and Hb were 0.694 and 0.749, while cut-off values were 49.5 ng/mL and 102.5 g/L, respectively. The sensitivity and specificity of NSE were 72.2% and 66.4%, respectively, while those of Hb were 72.2% and 74.2%, respectively. The result reveals that NSE and Hb represent promising significant risk factors of IOM for RCC patients. Notably, Hb is more reliable than NSE in distinguishing case of IOM from NIOM in patients with RCC.

## 1. Introduction

Renal cell carcinoma (RCC) is the most common kind of malignant tumor in the kidney. In addition, the development of macroscopic metastases arising from RCC represents the major cause of tumor-associated deaths [1]. Radical nephrectomy is the gold standard in the cure of RCC. But the survival rates of RCC remain unsatisfactory, even in cases with localized disease [2]. The estimated number of RCC stands at 90% of kidney cancers, while 80% of those are cases with clear cell histology. Uncommon cell malignancies include papillary, chromophobe, and collecting duct tumors [3]. Age and gender are strongly relevant to the risk of RCC, with the incidence of RCC being higher in the elderly population [4].

In adults, the most common form of intraocular malignancy is metastatic cancer [5]. Previous research has shown that choroidal metastases are the most common form of intraocular tumor [6], and the prognosis of these tumors depends on the primary site [7]. Visual impairment caused by metastatic cancers in the ocular is one of the most important factors affecting the life quality of patients [5]. Approximately 20-30% of RCC patients present with metastatic disease. Furthermore, the patients who suffer from advanced and metastatic disease are associated with lower survival rates [8]. Although metastases from RCC can occur at any anatomical site, they are most commonly observed in the lungs, bone, liver, and brain [9]. Cases with metastatic RCC (mRCC) are particularly associated with a poor prognosis if metastasis occurs at multiple sites or involves the bones or the liver [10]. Previous reports showed that RCC rarely metastasizes to the eye and that usual regions of intraocular metastasis (IOM) include the choroid, iris, and ciliary body [11]. The spread in ocular of RCC patients is thought to be achieved via the venous diffusion of neoplastic cells, as emboli, within the small choroidal vessels [12]. Though the eye is a less common site for migration, IOM is strongly related to RCC. Consequently, the early discovery and diagnosis of IOM and treatment in time are of great importance for RCC patients.

Ultrasound, magnetic resonance imaging (MRI), and computed tomography (CT) are predominant methodologies used in the diagnosis of RCC [13]. But there are some critical limitations associated with these imaging modalities, including cost and frequent exposure to radiation. Some serum tumor markers, such as neuron-specific enolase (NSE), carcinoembryonic antigen (CEA), alkaline phosphatase (ALP), and hemoglobin (Hb), are considered to be an important diagnostic and prognostic indicator for patients with renal cell carcinoma [14–17]. The abnormal expression of NSE is a risk factor for many neoplastic disorders, and it is closely related to the disease stage and prognosis [18]. The preoperative levels of Hb are also an independent prognostic indicator of cancer-related survival and overall survival in several carcinomas. However, the predictive and diagnostic value of the above serum tumor markers in metastatic renal cell carcinoma patients is still unclear, and the results are still controversial [19–21]. The prompt detection of IOM may significantly influence the choice of treatment against RCC. Consequently, there is an urgent need to investigate the indicators of IOM for RCC patients and determine clinically meaningful predictors.

In this retrospective research, the purpose was to determine possible risk factors for IOM by investigating a range of clinicopathological parameters and biomarkers in patients with RCC.

## 2. Materials and Methods

### 2.1. Study Design

**Figure 1:**
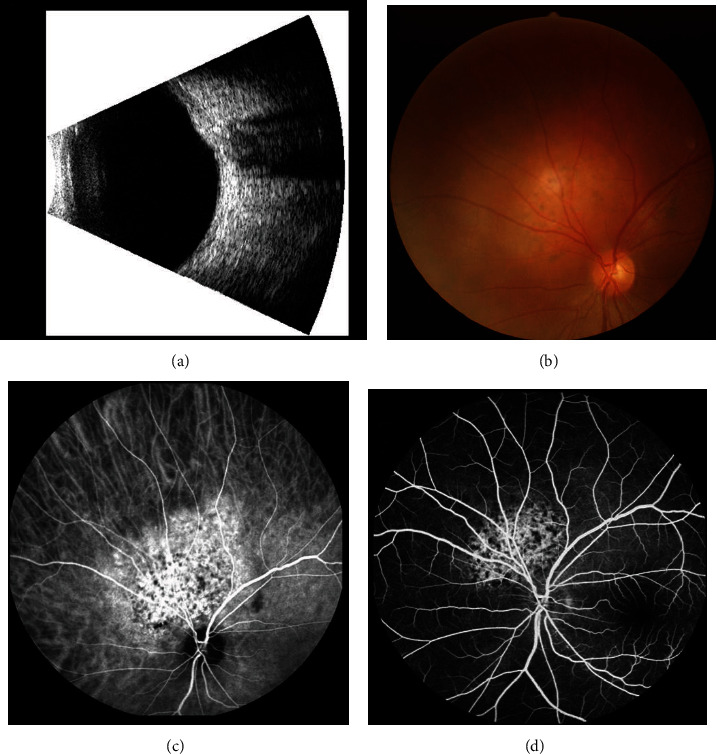
An example of RCC patients with IOM. Notes: (a) ophthalmic B-type ultrasound; (b) fundus photography; (c) indocyanine green angiography; and (d) fundus fluorescein angiography. Abbreviations: RCC: renal cell carcinoma; IOM: intraocular metastasis.

The retrospective research was conducted between May 1994 and May 2016 and involved a series of coherent patients who had been diagnosed with RCC in the First Affiliated Hospital of Nanchang University. For patients participating in the study, the diagnosis of primary RCC was confirmed via tissue pathological analysis obtained through needle biopsy or radical nephrectomy. The IOM patients were examined with ophthalmic B-type ultrasound, fundus photography, indocyanine green angiography (ICGA), and fundus fluorescein angiography (FFA) (Figure 1), and the diagnosis of IOM was confirmed using CT or MRI. This research ruled out the patients with primary ocular benign tumors, ocular malignancy, and secondary renal carcinoma. The study was approved by the ethics committee of the Hospital (Ethical code: CDYFY-20140214). Patients participating in the research received instructions with regard to the experimental design and supplied written informed consent.

### 2.2. Data Collection

For each participant, we retrospectively recorded a range of demographic and clinical characteristics, including gender, age at the time of diagnosis of the primary tumor, histopathological tumor subtype, sites of metastases, and treatments. We also retrospectively recorded the levels of a range of tumor biomarkers in the plasma, including NSE, alpha-fetoprotein (AFP), CEA, ALP, cancer antigen 125 (CA-125), cancer antigen 153 (CA-153), cancer antigen 199 (CA-199), calcium, and Hb. We subsequently analyzed the incidence of IOM and investigated data for the potential correlation between clinical parameters and IOM.

### 2.3. Statistical Analysis

The differences between RCC patients with IOM and RCC patients with non-IOM (NIOM) were assessed using the Chi-square test and Student *t* test. Binary logistic regression analysis was applied to determine the risk factors of IOM. In addition, the value in the diagnosis for RCC patients with IOM was evaluated using receiver operating characteristic (ROC) curve analysis, and area under the curve (AUC) values were figured to evaluate the precision for predicting IOM, the cut-off points were determined where the sensitivity and specificity are highest, which means that that value can classify whether the patient has disease best. All methods were carried out in the SPSS 17.0 software (IBM Corp, USA) and Excel 2010 software (Microsoft Corp, USA). *P* value <0.05 denoted statistical significance.

## 3. Results

### 3.1. Demographics and Clinical Characteristics

**Figure 2:**
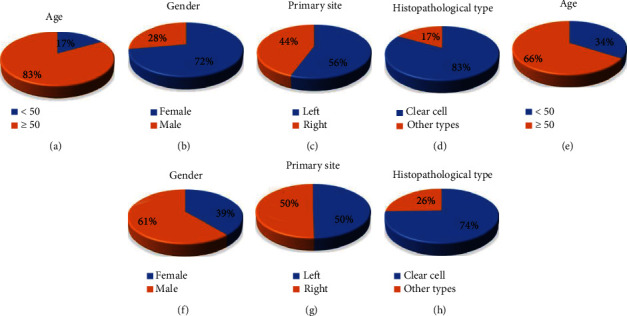
Clinical features of RCC patients with and without IOM. Notes: At the top are age (a), gender (b), primary site (c) and histopathological type (d) of IOM, the below are age (e), gender (f), primary site (g), and histopathological type (h) of NIOM. Abbreviations: RCC: renal cell carcinoma; IOM: intraocular metastasis; NIOM: nonintraocular metastasis.

**Figure 3:**
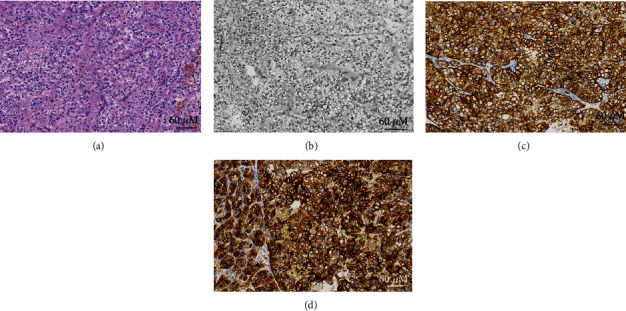
The HE and IHC staining images of IOM in RCC patients. Notes: (a) HE; (b) RCC; (c) CA9; and (d) CD10.

There are a total of 351 patients with RCC that were recruited in this study. Demographic data are presented in Table 1 and Figure 2. Typical HE and IHC staining images of specimens gathered from the IOM part of RCC are shown in Figure 3. More than half of the subjects were male (208 cases, 59.3%), and the main histopathological subtype was clear cell carcinoma (263 cases, 74.9%). Of the 351 patients, 18 were diagnosed with IOM. The mean age of patients in the IOM and NIOM groups was 59.83 ± 12.21 years and 56.02 ± 14.34 years, respectively. There are no significant differences in the age, primary tumor site, and histopathological subtype between the two groups (*P* > 0.05). But the difference in gender of the two groups was significant (*P* < 0.05). [17]

### 3.2. Clinical Features as the Risk Factors of IOM

Our analysis revealed that the levels of NSE were increased obviously (*P* < 0.05), whereas those of Hb were decreased obviously (*P* < 0.05) in the IOM group versus the NIOM group. However, the differences in the values of AFP, CEA, ALP, CA-125, CA-153, CA-199, or calcium between the two groups were not significant (Table 2). In order to exclude the gender difference as a potential bias, we further compared the values of NSE and Hb between males and females, and there were no significant differences between genders in both NSE and Hb (*P* > 0.05) (Table 3). Then binary logistic regression analysis showed that the values of NSE and Hb can be independent risk factors of IOM (Table 4).

### 3.3. Cut-off, AUC, Sensitivity, and Specificity Values for NSE and Hb Levels

**Figure 4:**
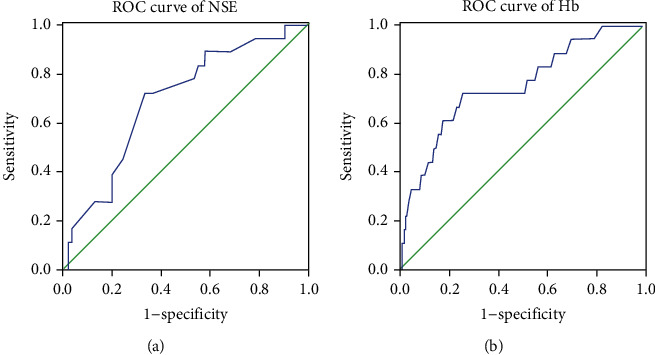
The ROC curves of risk factors in detecting IOM in RCC. Notes: (a) The ROC curve of NSE. The AUC is 0.694 (*P* = 0.006; 95% CI: 0.577-0.810); (b) The ROC curve of Hb. The AUC is 0.749 (*P* < 0.001; 95% CI: 0.627-0.871). Abbreviations: ROC: receiver operating characteristic; IOM: intraocular metastasis; NSE: neuron-specific enolase; Hb: hemoglobin; CI: confidence interval.

**Figure 5:**
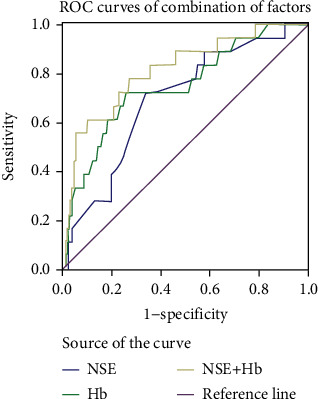
The ROC curves of combination of NSE and Hb. Notes: The AUC of the combination is 0.815 (*P* < 0.001; 95% CI: 0.708-0.921). Abbreviations: ROC: receiver operating characteristic; NSE: neuron-specific enolase; Hb: hemoglobin; CI: confidence interval.

The AUC value for NSE was 0.694, and the associated sensitivity and specificity values in predicting IOM were 72.2% and 66.4%, respectively (Figures 4 and 5; Table 5). The AUC value of Hb was 0.749, and the associated sensitivity and specificity values in predicting IOM were 72.2% and 74.2%, respectively. In addition, cut-off values of NSE and Hb were 49.5 ng/mL and 102.5 g/L, respectively. We also found that the combination of NSE and Hb data exhibited higher AUC (0.815) and specificity (90.1%) values. All data were statistically significant (*P* < 0.05).

## 4. Discussion

Thus far, previous studies have reported IOM in patients with nonsmall cell lung carcinoma [22], esophageal cancer [23], thyroid cancer [24], gastric adenocarcinoma [25], breast cancer [7], choriocarcinoma [26], colon adenocarcinoma [27], and prostatic adenocarcinoma [28] (Table 6). IOM in patients with RCC is uncommon and may occur years off treatment of primary cancer. Therefore, IOM should not be neglected in follow-up surveillance of patients who have received treatment for RCC [29]. Intraocular mRCC within the choroid is the most common form of IOM and may exhibit a similar clinical appearance to that of uveal melanoma [30]. Metastases to the iris and the ciliary body are comparatively uncommon in patients with RCC and tend not to be detected in clinical practice except existing a history or clinical certification of systemic malignancy [31]. Certain treatments against IOM may be performed for esthetical or functional considerations, even in cases where there is no cure [32]. Because many of these patients have a more severe systemic disease, treatment options for intraocular lesions are limited, and the effect on ocular may be severe with restricted advantage for visual recovery [33]. Though the initial histopathology of RCC can generally be transferred to distant locations of metastasis, the differentiation of metastatic tumors can be worse or exhibit a diverse range of morphological characteristics [34]. Therefore, the identification of reliable predictive factors for IOM in patients with RCC is critical in allowing timely intervention, which may prevent or delay the course of RCC. Hence, it is important to distinguish particular patients who may benefit from complementary forms of treatment.

NSE, known as a highly specific marker of neurocytes and peripheral neuroendocrine cells, is a cell-specific isoenzyme of the glycolytic enzyme enolase [15]. Enolase exists in three forms, namely *α*, *β*, and *γ*. The *γ* form is commonly referred to as NSE because it is specific to neurons [35]. Due to organ-specific localization, the levels of NSE in serum and ncurolymph are usually increased in abnormalities involving neural damage. Thus, NSE can be used to evaluate the degree of neural injury in different situations [36]. Currently, NSE is the only suggested prognostic indicator for hypoxic brain injury following cardiopulmonary resuscitation [37]. Furthermore, NSE is useful in the monitoring of patients with neuroendocrine tumors [38]. In addition, NSE is generally recognized as a reliable biomarker in the diagnosis and prognosis of small cell lung carcinoma [39]. NSE is particularly useful in the diagnosis of malignant tumors, and is expressed in a number of RCC subtypes, particularly clear cell RCC (ccRCC) [40]. Moreover, almost all cases showing a morphological presentation consistent with ccRCC also exhibited high expression of NSE [41]. Following treatment for RCC, the levels of NSE in the serum decrease. Therefore, according to previous research, NSE may be a useful marker during periods of surveillance for RCC [42, 43]. Increased levels of NSE in the serum have been detected in all stages of neuroblastoma. However, increased levels of NSE are more notable in cases involving widespread and metastatic disease [15]. In line with the findings of previous studies, we detected increased values of NSE in the serum of RCC patients, as well as an obvious difference in the levels of NSE between patients with and without IOM. We determined that the cut-off value of NSE was 49.5 ng/mL and that serum NSE was an independent risk factor of IOM. This finding indicates that serum NSE levels >49.5 ng/mL may be a risk factor in predicting IOM for RCC patients.

Hb is a useful prognostic factor of survival. The preoperative levels of Hb are an independent unfavorable prognostic indicator of cancer-related survival and overall survival (OS) in patients experiencing radical cystectomy for transitional cell carcinoma [19]. The preoperative levels of Hb can also predict poor survival in patients suffering from upper urinary tract urothelial carcinoma [20]. In addition, Hb has been identified as a part of bone marrow that is particularly concerned with the metastasis of prostate cancer to the bones [21]. In patients with mRCC, Hb variability is an independent cause of deaths and may be related to OS [44]. Indeed, previous research has shown that mRCC patients receiving treatment with tyrosine kinase inhibitors, who exhibit increased levels of Hb, were linked to longer OS and progression-free survival [45]. Furthermore, mRCC patients showing an early increase in the levels of Hb during treatment with axitinib have been related to an obvious improvement in clinical outcome [46]. The occurrence of anemia in patients with ccRCC increases the risk of death by causes other than RCC [47]. Anemia may also result in tumor hypoxia, which plays a detrimental factor for cure and prognosis [44]. In the research, we demonstrated that the level of Hb may be an independent risk factor of IOM in RCC patients. Moreover, the cut-off value for Hb was 102.5 g/L. Therefore, the present findings suggest that serum Hb levels <102.5 g/L may assist in identifying populations of RCC patients who are significantly more likely to develop IOM.

Interestingly, our current analysis identified the levels of NSE and Hb as independent risk factors in predicting IOM for RCC patients. However, there were certain limitations in the research. Firstly, this is a retrospective research, while some information was missing in the case history. Secondly, all medical records used in our analysis were collected from a single medical institution. This practice may have potentially introduced some bias. Thirdly, only 18 patients were diagnosed with IOM among 351 RCC patients in our study, which might need more data support in the future to make our conclusion more statistically reliable. Lastly, our study merely demonstrated correlations between alterations in the levels of NSE and Hb and IOM in patients with RCC. Our current data do not allow us to determine the mechanisms through which IOM results in the observed changes in the levels of NSE and Hb. Consequently, a prospective, multicenter study is warranted to verify the present results.

## 5. Conclusion

We showed that the levels of NSE and Hb are promising significant risk factors of IOM in patients with RCC. Furthermore, the combination of NSE and Hb data exhibited higher specificity. Therefore, we recommend intensive monitoring and radiological examinations (i.e., head CT or MRI) in all newly diagnosed RCC patients with NSE levels >49.5 ng/mL or Hb levels <102.5 g/L.

## Figures and Tables

**Table 1:** Clinical features of RCC patients.

Patient characteristics	IOM group (%)	NIOM group (%)	Total numbers of patients (%)	*P* value
*Age (years)^a^*	59.83 ± 12.21	56.02 ± 14.34		0.269
<50	3 (16.7%)	114 (34.2%)	117 (33.3%)	
≥50	15 (83.3%)	219 (65.8%)	234 (66.7%)	
*Gender^b^*				0.005
Female	13 (72.2%)	130 (39.0%)	143 (40.7%)	
Male	5 (27.8%)	203 (61%)	208 (59.3%)	
*Primary site^b^*				0.637
Left	10 (55.6%)	166 (49.8%)	176 (50.1%)	
Right	8 (44.4%)	167 (50.2%)	175 (49.9%)	
Bilateral	0	0		
*Histopathological type^b^*				0.572
Clear cell	15 (83.3%)	248 (74.5%)	263 (74.9%)	
Other types	3 (16.7%)	85 (25.5%)	88 (25.1%)	

Notes: ^a^Student *t* test was applied to analysis. ^b^Chi-square test was applied to analysis. *P* value <0.05 represented statistically significant. Abbreviations: IOM: intraocular metastasis; NIOM: nonintraocular metastasis.

**Table 2:** Differences in the concentration of various tumor biomarkers between RCC patients with and without IOM.

Clinical features	IOM group	NIOM group	*t*	*P* value
NSE	53.50 ± 14.97	41.36 ± 17.66	2.86	0.004
AFP	3.68 ± 1.12	4.90 ± 3.74	-1.382	0.168
CEA	35.38 ± 95.41	3.80 ± 3.17	1.404	0.178
CA-125	41.85 ± 25.67	28.57 ± 111.92	0.502	0.616
CA-153	24.48 ± 16.38	22.56 ± 10.34	0.492	0.629
CA-199	18.88 ± 12.21	15.41 ± 14.05	1.026	0.306
ALP	337.50 ± 644.05	81.56 ± 60.44	1.686	0.110
Hb	91.50 ± 28.12	117.32 ± 25.17	-4.213	<0.001
Calcium	2.40 ± 0.43	2.29 ± 0.25	1.084	0.293

Notes: *P* < 0.05 represented statistically significant. Abbreviations: IOM: intraocular metastasis; NIOM: nonintraocular metastasis; NSE: neuron-specific enolase; AFP: alpha-fetoprotein; CEA: carcinoembryonic antigen; ALP: alkaline phosphatase; Hb: hemoglobin.

**Table 3:** Differences in the concentration of various tumor biomarkers between male and female.

Clinical features	Male group	Female group	t	*P* value
NSE	40.67 ± 17.54	43.90 ± 18.02	1.670	0.096
Hb	117.12 ± 26.64	114.33 ± 24.61	-0.994	0.321

Notes: *P* < 0.05 represented statistically significant. Abbreviations: NSE: neuron-specific enolase; Hb: hemoglobin.

**Table 4:** The binary logistic regression results.

Factors	B	OR	OR (95% CI)	*P* value
NSE	0.043	1.044	1.011-1.078	0.008
HB	-0.035	0.966	0.948-0.983	<0.001

Notes: *P* < 0.05 represented statistically significant. Abbreviations: B: coefficient of regression; OR: odds ratio; CI: confidence interval; NSE: neuron-specific enolase; Hb: hemoglobin.

**Table 5:** The ROC results of risk factors for predicting IOM in RCC patients.

	Cut-off value	Sensitivity (%)	Specificity (%)	AUC	CI (95%)	*P* value
NSE	0.386	0.722	0.664	0.694	0.577-0.810	0.006
HB	0.464	0.722	0.742	0.749	0.627-0.871	<0.001
NSE+Hb		0.611	0.901	0.815	0.708-0.921	<0.001

Notes: *P* < 0.05 represented statistically significant. Abbreviations: AUC: area under the curve; CI: confidence interval; NSE: neuron-specific enolase; Hb: hemoglobin.

**Table 6:** Studies on the IOM from different cancers.

Author	Year	Diseases with IOM
Singh, A., et al. [14]	2010	Nonsmall cell lung cancer
Lv, D., et al. [15]	2015	Esophageal carcinoma
Ozpacaci, T., et al. [16]	2012	Thyroid cancer
Kim, S. Y., et al. [17]	2018	Gastric adenocarcinoma
Demirci et al. [7]	2003	Breast cancer
Hazan, A., et al. [18]	2014	Choriocarcinoma
Nookala, R., et al. [19]	2016	Colon adenocarcinoma
Albadainah, F., et al. [20]	2015	Prostatic adenocarcinoma

Notes: The table summed up studies on IOM from different types of cancer. Abbreviations: IOM: intraocular metastasis.

## Data Availability

The datasets used and/or analyzed during the current study are available from the corresponding author on reasonable request.
